# Reassessing Antibiotic Duration in Dental Infections: A Narrative Review and Marginal Benefit–Harm Framework with a Clinical Checklist

**DOI:** 10.3390/antibiotics15090924

**Published:** 2026-09-18

**Authors:** Jelena Roganović, Vuk Roganović

**Affiliations:** 1 Department of Pharmacology in Dentistry, Faculty of Dental Medicine, University of Belgrade, Subotića 8, 11000 Belgrade, Serbia; 2 Faculty of Economics and Bussiness, University of Belgrade, 11000 Belgrade, Serbia; vukroganovic2004@gmail.com

**Keywords:** antibiotic duration, dental infections, antimicrobial stewardship, clinically sufficient antibiotic exposure, marginal benefit–harm, source control, pharmacokinetics/pharmacodynamics, antibiotic reassessment

## Abstract

Antibiotic duration is a central question in antimicrobial stewardship, yet dental prescribing often remains anchored to fixed five- or seven-day courses despite the source-control-dependent nature of many odontogenic infections. Current dental guidance supports short courses and clinical reassessment but provides limited structure for deciding whether continued antibiotic exposure remains justified once treatment has begun. This narrative review aimed to integrate clinical, pharmacokinetic/pharmacodynamic, dental, and ethical evidence within a marginal benefit–harm framework and to translate that framework into a practical reassessment checklist. A targeted narrative search identified 147 records from PubMed/MEDLINE, Google Scholar, professional guidance documents, and reference chaining; 52 references were selected across four evidence streams: studies comparing shorter and longer antibiotic courses, dental evidence and guidance on odontogenic infections, PK/PD literature on antimicrobial exposure, and ethics and stewardship literature on proportionality, antimicrobial resistance, and public-health responsibility. The review conceptualizes clinically sufficient antibiotic exposure as the patient-specific decision point at which the expected marginal clinical benefit of further antibiotic treatment no longer clearly exceeds its expected incremental harm. This is not defined by a fixed number of treatment days and may change according to source control, clinical trajectory, antimicrobial adequacy, infection severity, anatomical risk, and host factors. On this basis, the proposed checklist operationalizes the marginal model by structuring reassessment of infection severity, anatomical involvement, host-related risk modifiers, and the marginal benefit–harm balance of further treatment. Rather than promoting automatic shortening, the framework supports continuation only when a specific unresolved clinical factor provides a plausible justification for additional exposure. This approach reframes antibiotic duration in dental infections from a predetermined calendar course to a patient-specific judgment about whether further exposure remains clinically and ethically justified.

## 1. Introduction

Antibiotic duration remains one of the most clinically important and ethically sensitive questions in antimicrobial stewardship. In medicine, the long-standing instruction to “complete the course” has increasingly been challenged by randomized trials showing that shorter regimens are often non-inferior to longer ones in selected infections [1,2]. At the same time, excessive antibiotic duration has been framed as clinically as well as ethically problematic because it exposes patients and populations to avoidable harm when shorter courses preserve clinical resolution [3]. This argument is persuasive, but it should not be simplified into a new rule that “shorter is always better.” The more clinically useful question is not whether antibiotic therapy should always be shorter, but whether the patient has received enough active antibiotic exposure to secure clinical resolution. This is particularly relevant in dentistry, where resolution of odontogenic infection usually depends on definitive dental treatment and source control, yet antibiotic therapy is still often prescribed as a fixed five- or seven-day course [4,5,6]. These recommendations should not be regarded as universally applicable durations for all dental infections, since these findings may not apply to patients with poor source control, deep or spreading infection, osteomyelitis, severe immunosuppression, or other high-risk conditions [5,6,7].

Current dental guidance supports short treatment durations and appropriate indications but provides less explicit support for deciding whether antibiotics should be continued once treatment has begun. This creates a practical gap between recommending short courses and justifying continuation in an individual patient. To address this gap, this narrative review applies a marginal benefit–harm framework, adapted from marginal analysis, to integrate clinical, pharmacological, dental, and ethical evidence into an explicit reassessment rule: whether further antibiotic exposure is expected to provide enough additional benefit to justify its additional harms. Within this framework, *clinically sufficient antibiotic exposure* is conceptualized as the patient-specific decision point at which the expected additional benefit of continued treatment no longer clearly exceeds its incremental harm. This point is not defined by a fixed number of treatment days, but varies according to infection severity, anatomical involvement, and host-related risk modifiers. The framework is then translated into a structured checklist for decisions about continuation or discontinuation.

## 2. Narrative Review Approach

This narrative review was designed as a conceptual synthesis, and not as exhaustive coverage of all available literature on antibiotic use in medical and/or dental practice. The search was structured around four predefined evidence domains: (1) medical trials and systematic reviews comparing shorter and longer antibiotic treatment courses; (2) dental studies, reviews, and professional guidelines addressing odontogenic infections, source control, antibiotic indications, and treatment duration; (3) pharmacokinetic/pharmacodynamic literature relevant to antimicrobial exposure, tissue penetration, and treatment adequacy; and (4) ethics and antimicrobial-stewardship literature addressing proportionality, antimicrobial resistance, avoidable treatment burden, and public-health responsibility. Searches were conducted in PubMed/MEDLINE and Google Scholar. The final search was conducted in July 2026. The primary search combinations included “antibiotic duration” AND (“dental infection” OR “odontogenic infection”), “short-course antibiotics” AND dentistry, “source control” AND (“dental infection” OR “odontogenic infection”), (“pharmacokinetics” OR “pharmacodynamics” OR “antimicrobial exposure”) AND antibiotic duration, and (“antibiotic stewardship” OR “antimicrobial resistance”) AND (“ethics” OR “proportionality”); additional dentistry-specific combinations included terms related to endodontic infection, periodontitis, maxillofacial infection, and dental antibiotic prescribing. Search terms were combined iteratively within each of the four predefined evidence domains and adapted as needed to the respective database.

Eligible sources included randomized and non-randomized clinical studies, systematic reviews, scoping reviews, narrative reviews when directly relevant to the conceptual question, professional society guidelines, public-health guidance, and ethics or stewardship literature addressing the continuation or duration of antimicrobial therapy. The search was restricted to publications in English, with no restriction on publication date. Sources were excluded if they were unrelated to treatment duration or reassessment, focused solely on in vitro susceptibility or laboratory microbiology without implications for clinical prescribing duration, involved non-human or veterinary populations, or duplicated a point already supported by a more directly relevant or higher-level source. No study was excluded solely because it did not support the proposed framework.

The search identified 147 records. After removal of 31 duplicates, 116 records were screened by title and abstract. Forty records were excluded at this stage because they did not address antibiotic duration, reassessment, or clinically relevant determinants of continued treatment. Seventy-six full-text records were assessed, of which 24 were excluded because they provided information already represented by more directly relevant sources or did not contribute materially to one of the four predefined evidence domains. The final evidence synthesis therefore comprised 52 references. One additional contemporary reference on infective endocarditis prevention was incorporated during manuscript revision in response to peer-review feedback, bringing the total reference list to 53. The final references were chosen to provide balanced conceptual coverage across the four predefined themes (Table 1), as well as to develop the proposed checklist for reassessing antibiotic duration in dental infection treatment as a structured, evidence-informed tool for clinical reasoning and not a substitute for clinician judgment.

## 3. Evidence Informing Antibiotic-Duration Reassessment

### 3.1. Indications, Duration, and Stewardship Considerations in Dental Antibiotic Prescribing

Indications for antibiotic use in dentistry can be broadly divided into therapeutic and prophylactic use. For therapeutic prescribing, systemic antibiotics should generally be considered an adjunct to, rather than a substitute for, definitive operative treatment. In endodontic and odontogenic infections, antibiotics are indicated when infection extends beyond the local dental focus or is accompanied by systemic involvement, including cellulitis, fascial-space spread, fever, malaise, trismus, lymphadenopathy, dysphagia, or other signs of progressive infection [7,15]. The 2019 American Dental Association (ADA) guideline, developed using GRADE methodology, recommends against systemic antibiotics for most pulpal and periapical conditions in immunocompetent adults when definitive conservative dental treatment can be provided, and reserves antibiotic therapy primarily for cases with systemic involvement or when adequate local management cannot be achieved [7]. Similarly, the European Society of Endodontology emphasizes pulpectomy, drainage, extraction, or other source-control procedures as the primary intervention and limits systemic antibiotic use to infections with systemic or progressive spread [15]. Accordingly, conditions such as irreversible pulpitis, localized periapical infection with adequate drainage, infected root canals without systemic involvement, and uncomplicated extractions generally do not justify systemic antibiotic therapy, although unnecessary prescribing for these indications remains common in practice [7,15,16].

Periodontal therapy requires a similarly selective approach. Mechanical disruption of the subgingival biofilm remains the primary treatment, while systemic antibiotics—most commonly amoxicillin combined with metronidazole—may provide additional benefit in selected patients with severe stage III/IV or grade C periodontitis. Their routine use in less severe periodontal disease is not supported because the expected incremental clinical benefit is small relative to the ecological and adverse-effect burden [17].

Prophylactic indications are considerably narrower. Infective endocarditis prophylaxis is restricted to patients at highest cardiac risk, including those with prosthetic cardiac valves or prosthetic material used for valve repair, a previous history of infective endocarditis, selected congenital heart diseases, and cardiac transplantation complicated by valvular disease, when they undergo invasive dental procedures associated with manipulation of gingival tissue, the periapical region, or perforation of the oral mucosa [18]. Prophylactic antibiotics in severely immunocompromised individuals may be considered on an individualized basis according to the underlying condition, degree of immunosuppression, planned procedure, and specialist advice. Outside these clearly defined groups, routine prophylaxis is generally not supported, and observational evidence suggests that a substantial proportion of prophylactic prescriptions before dental procedures are discordant with current recommendations [19].

Duration is equally important. Current dental stewardship recommendations generally favor the shortest effective course. The 2026 American Dental Association Clinical Practice Statement reinforces a source-control-centered and reassessment-based approach to dental antibiotic use. Definitive dental treatment remains the primary intervention for localized odontogenic infection, while systemic antibiotics should be reserved for systemic involvement or spreading infection, prescribed for the shortest effective duration, and discontinued within 24–48 h after resolution of systemic signs and symptoms [16]. Evidence from systematic reviews of odontogenic and maxillofacial infections suggests that courses of approximately 3–5 days are frequently sufficient when incision, drainage, extraction, or other definitive source-control procedures are effective, whereas longer treatment may be justified in deep fascial-space infection, osteomyelitis, severe anatomical spread, persistent systemic involvement, inadequate drainage, immunosuppression, or other clinical features associated with a higher risk of treatment failure or complications [20,21,22]. Thus, current guidelines provide recommended durations and reassessment points, but they do not explicitly structure how clinicians should integrate source control, clinical trajectory, antimicrobial adequacy, infection severity, host factors, and treatment-related harms when deciding whether further exposure remains justified in an individual patient. The aim of the proposed checklist is to convert guideline-based reassessment into a structured continuation decision. By requiring clinicians to identify the specific unresolved factor that still justifies additional antibiotic exposure, the checklist makes continuation or discontinuation more explicit, individualized, and clinically accountable.

The oral cavity is especially relevant to antimicrobial-resistance stewardship because it contains one of the most diverse microbial ecosystems in the human body. Distinct ecological niches—including saliva, the tongue, supragingival and subgingival plaque, mucosal surfaces, periodontal pockets, prostheses, and implant-associated surfaces—support dense microbial communities with substantial opportunities for horizontal gene transfer [23]. The collective pool of antimicrobial-resistance genes within these communities constitutes part of the oral resistome, which may serve as a reservoir for resistance determinants that can potentially disseminate within and beyond the oral microbiome [23,24].

Biofilm formation further amplifies this concern. Oral microorganisms form structured biofilms on teeth, mucosal surfaces, restorations, dentures, implants, orthodontic appliances, and dental-unit waterlines [25,26]. Consequently, repeated or unnecessary antimicrobial exposure may not simply affect the treated pathogen but also exert selective pressure across a complex microbial ecosystem. Resistance genes involving tetracyclines, macrolides, beta-lactams, and multidrug-resistance mechanisms have been identified within oral microbial communities, often associated with mobile genetic elements capable of facilitating dissemination [24]. A substantial proportion of dental prescriptions remain unnecessary, broader in spectrum than required, or longer than clinically justified [21,27]. The consequences of unnecessary exposure therefore extend beyond classical treatment failure. Antibiotics may cause immediate patient-level harms, including hypersensitivity reactions, gastrointestinal adverse effects, drug interactions, and *Clostridioides difficile* infection, while also disrupting commensal microbiota and promoting dysbiosis. At the ecological level, repeated exposure can select resistant organisms and alter the oral resistome, with potential implications for both individual and population-level antimicrobial resistance [24,25,26]. This is why operative source control is central to dental stewardship: effective drainage, endodontic treatment, extraction, periodontal debridement, and other local interventions reduce the need for prolonged systemic antimicrobial exposure and thereby reduce opportunities for resistance selection [7,16,25].

### 3.2. Clinically Sufficient Antibiotic Exposure: Concept and Pharmacological Rationale

Based on the intention to bring together several already recognized principles: shortest effective duration, adequate antimicrobial exposure, dental source control, early clinical response, infection severity, host-related modifiers, and avoidance of unnecessary antibiotic harm, this review introduces the term clinically sufficient antibiotic exposure to describe the patient-specific decision point at which the expected additional benefit of further antibiotic treatment no longer clearly exceeds its incremental harms. It is an evidence-informed clinical reasoning construct, not a validated endpoint or universal duration rule. Its purpose is to make explicit a question that is often left implicit: after source control and early clinical response have been reassessed, what unresolved factor still justifies further antibiotic exposure? The term sufficient emphasizes that exposure should be enough to support clinical resolution, but not exceed what is necessary. The term *exposure* shifts attention beyond the number of treatment days to the conditions that determine antibiotic effectiveness, including dose, route, dosing interval, adherence, tissue penetration, likely susceptibility, source control, infection severity, host factors, and clinical trajectory. Thus, clinically sufficient antibiotic exposure represents a patient-specific, reassessment-based decision point, not a predetermined duration.

From a pharmacodynamic perspective, antibiotic efficacy depends on achieving adequate exposure against a susceptible pathogen rather than on calendar duration alone [37]. For β-lactams, efficacy is primarily related to the time that free drug concentrations remain above the MIC, whereas concentration-dependent agents such as aminoglycosides and fluoroquinolones are better described by peak/MIC or AUC/MIC relationships [38,39]. Pharmacokinetic factors—including absorption, distribution, tissue penetration, perfusion, metabolism, and clearance—determine whether effective exposure is achieved at the site of infection [40].

Accordingly, clinically sufficient antibiotic exposure is proposed as a patient-specific reassessment concept rather than a fixed treatment duration. Shorter therapy may be sufficient when the antibiotic is appropriate, reaches the infection site adequately, the pathogen is susceptible, source control is achieved, and the patient is clinically improving. In contrast, poor penetration, resistant pathogens, abscesses, biofilm, necrotic tissue, severe infection, impaired immunity, or inadequate source control may justify longer treatment [14,31,32,37,38,39,40]. The relevant question is therefore not simply “How many days?” but whether sufficient antibiotic exposure has been achieved for this patient, infection site, pathogen, and clinical response.

This is particularly relevant in dentistry, where pus, necrotic tissue, abscess cavities, poor perfusion, biofilm, and infected pulp may limit antibiotic effectiveness [5,6,15,30,31,32]. If the source remains untreated, prolonging therapy may offer little additional benefit; after drainage, extraction, endodontic treatment, or debridement, sufficient exposure may be reached earlier. The proposed framework therefore supports neither automatic completion of fixed courses nor automatic shortening, but structured reassessment of whether further antibiotic exposure remains clinically and ethically justified.

### 3.3. Clinical Trials in Medicine and Dentistry Regarding Antibiotic Treatment Duration

Clinical trials in medicine provide the strongest evidence that many traditional antibiotic durations are longer than necessary. In community-acquired pneumonia, stopping antibiotics after three days in clinically stable patients was not inferior to continuing therapy for eight days [1]. In ventilator-associated pneumonia, eight days of therapy was comparable to fifteen days in many patients, although pathogen-specific relapse risk required caution [2]. In complicated intra-abdominal infection after adequate source control, the STOP-IT trial showed that approximately four days of antibiotics was comparable to longer therapy based on resolution of physiological abnormalities [8]. In uncomplicated Gram-negative bloodstream infection, seven days was non-inferior to fourteen days [9], with similar findings reported for Enterobacterales bloodstream infections [10]. C-reactive protein-guided therapy, seven-day therapy, and fourteen-day therapy have also been compared in uncomplicated Gram-negative bacteremia [11]. More recently, the BALANCE group of investigators reported that seven days was non-inferior to fourteen days for bloodstream infections [12]; shorter- and longer-duration antibiotics show similar efficacy, with consistent effects observed across age groups and bacterial types [13]. Together, these studies support the stewardship principle that prolonged antibiotic therapy is often unnecessary once adequate infection control has been achieved. However, they should not be interpreted as evidence that shorter therapy is appropriate for all patients or all infections, because many trials exclude or underrepresent patients with factors associated with a higher risk of treatment failure or complications, including severe immunosuppression, uncontrolled source of infection, deep-seated infection, prosthetic material, extensive biofilm, necrosis, abscess formation, hemodynamic instability, or highly resistant organisms. Moreover, randomized trials in pneumonia, intra-abdominal infection, and bloodstream infection provide broader proof-of-principle that shorter antibiotic courses may be non-inferior to longer courses when infection control is adequate and the patient is clinically stable [1,2,8,9,10,11,12,13]. These studies support the general rationale for reassessment but should not be interpreted as direct evidence for specific antibiotic durations in dental infections.

Dentistry adds a distinctive clinical context to this debate. While in medicine, duration usually depends on systemic infection, in dentistry, duration depends simultaneously on: source control, anatomical accessibility, biofilm, mechanical treatment, PK/PD and patient improvement. Namely, odontogenic infections differ from many medical infections because the source is often anatomically accessible. The infected tooth, necrotic pulp, periodontal pocket, abscess cavity, or fascial-space collection can often be treated directly through drainage, endodontic treatment, extraction, or surgical management [6,7,15,16]. In these conditions, systemic antibiotics are usually adjunctive to source control rather than definitive therapy alone [4,5,6], closely linking the duration question to source control and clinical response. On the other side, studies still show fixed-course habits and inappropriate prescribing among dental clinicians. Notably, the ideal duration of antibiotic treatment in many acute infections has been described as 3–7 days [4]. Furthermore, evidence linked duration more directly to surgical treatment, noting that short antibiotic courses may be sufficient after removal of the infectious source and that longer courses have not shown clear additional benefit [5,6]. In adults with dental infections in outpatient settings, a systematic review found only one small randomized controlled trial that met the inclusion criteria and, by comparing three-day versus seven-day amoxicillin in adults with odontogenic infection requiring extraction, found no significant difference in participant-reported pain or clinical wound healing [14]. However, the conclusions were limited because all participants started amoxicillin two days before extraction, which is not typical clinical practice [14]. Evidence from endodontic infections further supports reassessment rather than automatic completion of a fixed course. Drobac et al. found that 55.7% of surveyed Serbian dentists prescribed a 5-day antibiotic course for endodontic infections and that antibiotics were sometimes prescribed when not indicated, suggesting that fixed-duration habits and inappropriate indications remain common in dental practice [28]. Parirokh et al. evaluated antibiotic therapy in endodontic infections and reported that patients were monitored after treatment initiation, with antibiotic change considered if no recovery was seen after 24–48 h. Their findings also showed that drainage combined with amoxicillin was associated with faster recovery than antibiotics alone, reinforcing that duration decisions should be linked to monitoring and local intervention [29]. Recent endodontic review evidence is consistent with this restrictive approach and short courses to limit resistance [30]. Two recent systematic reviews have specifically examined antibiotic duration in odontogenic infections. Ribeiro et al. synthesized eight primary studies, largely emphasizing randomized evidence, and found that 3–5-day regimens may be effective when combined with surgical intervention, while calling for further randomized trials [31]. Alhudaithi et al. reviewed nine studies of maxillofacial space infections, including randomized, prospective, and retrospective designs, and reported that 2–5-day courses after prompt incision and drainage were generally effective, although the underlying evidence showed methodological limitations and variable risk of bias [32]. In periodontal infections, antibiotics are used as adjuncts to scaling and root planing or subgingival instrumentation in selected severe cases. The central intervention is mechanical disruption of the subgingival biofilm. Winkler et al. showed that systemic antibiotics in periodontitis can be targeted using patient-level criteria such as age and disease severity or microbiological findings, and that younger individuals with severe periodontitis appeared to benefit most from adjunctive antibiotics [33]. This supports a selective rather than routine approach to antibiotic use in periodontal therapy. Fermiano et al. add a further periodontal distinction by focusing not on total duration, but on the timing of systemic antibiotics relative to scaling and root planing. In patients with stage III/IV periodontitis, early and late administration of metronidazole plus amoxicillin produced comparable clinical improvements after one year, but early administration promoted faster and more favorable subgingival microbial reorganization [34]. This suggests that, in periodontal therapy, antibiotic effectiveness depends not only on the number of days prescribed, but also on coordination between antimicrobial exposure and mechanical biofilm disruption. Thus, while in acute odontogenic and endodontic infections, the key determinant is source control through drainage, endodontic treatment, extraction, or surgical management, in periodontitis, the key determinant is mechanical biofilm disruption and careful selection of patients most likely to benefit from adjunctive systemic antibiotics. Across both contexts, the remaining gap is not simply whether antibiotic courses should be shorter, but how clinicians should decide at reassessment whether further antibiotic exposure remains justified. Dental evidence and professional guidance support a source-control-centered and reassessment-based approach to antibiotic use. In odontogenic, endodontic, periodontal, and maxillofacial infections, systemic antibiotics are generally adjunctive to definitive local treatment, and available dental studies and guidelines support short, individualized courses when source control is achieved and clinical improvement is evident [4,5,6,7,14,15,28,29,30,31,32,33,34,35]. Even contemporary infective endocarditis prevention supports steps away from prolonged or precautionary antibiotic use toward risk-stratified prophylaxis, oral-health optimization, and multidisciplinary management [36]. Because high-quality duration trials in dentistry remain limited, specific dental treatment decisions should be based primarily on dentistry-specific evidence and guidance rather than directly extrapolated from medical duration trials. The CDC dental checklist, based on ADA guidance, advises clinicians to prioritize definitive conservative dental treatment, avoid antibiotics when definitive dental treatment is available for most dental conditions, prescribe antibiotics when systemic involvement such as fever or malaise is present, use the shortest effective duration of three to seven days when antibiotics are needed, follow up after three days, and discontinue antibiotics 24 h after complete resolution of systemic signs and symptoms [35,41]. This recommendation already moves dental prescribing away from fixed-duration habits and toward reassessment, and the presently proposed Checklist for reassessing antibiotic duration addresses the remaining gap by operationalizing this reassessment. Thus, the evidence summarized above represents established clinical and stewardship principles. The marginal benefit–harm framework proposed in this review is not itself an established guideline; it is a conceptual model that integrates these principles into an explicit decision about whether continued antibiotic exposure remains justified.

### 3.4. Ethical Principles Guiding Antibiotic Duration Decisions

In odontogenic infections, antibiotic duration is both a clinical and ethical decision [3,42,43]. The aim is not simply shorter treatment, but the clinically sufficient exposure needed to support clinical resolution while avoiding unnecessary continuation.

Beneficence requires adequate treatment when antibiotics are indicated. In infections with systemic involvement, spreading cellulitis, deep-space infection, severe progression, or increased host-related factors associated with a higher risk of complications or treatment failure, stopping antibiotics too early may cause avoidable harm, while longer duration is justified when infection remains uncontrolled [3,44]. Non-maleficence requires avoiding unnecessary antibiotic harm, including adverse reactions, drug interactions, microbiome disruption, Clostridioides difficile infection, and resistance selection. Each additional day should have a clear clinical purpose. Once source control is achieved and systemic signs have resolved or are regressing, continued therapy becomes harder to justify [45,46].

Proportionality means that duration should match infection severity, source-control status, antimicrobial suitability, tissue penetration, host-related risk modifiers, and clinical response [47]. Fixed five- or seven-day courses should not replace reassessment. Longer courses remain proportionate when deep-space involvement, osteomyelitis, persistent fever, poor source control, necrosis, abscess persistence, or impaired host response is present [48]. Justice recognizes antibiotics as shared social resources [29,30]. Unnecessary antibiotic days in dental practice increase selection pressure and may reduce future treatment options, making avoidable continuation ethically relevant beyond the individual patient [45,49,50]. Ecological responsibility extends this concern to the microbiome, community, environment, and future generations [51]. Prolonged exposure can disturb microbiota and contribute to wider antimicrobial resistance pressures, especially when definitive dental treatment, rather than continued antibiotics alone, is required [45,51,52].

## 4. Conceptual Synthesis and Marginal Benefit–Harm Framework

The recurring determinants of antibiotic continuation identified across the four evidence streams were integrated within a marginal benefit–harm framework. Marginal analysis, originating in economics, evaluates whether one additional unit of an intervention provides enough additional benefit to justify its additional cost or burden; in health care, the same logic can be applied without monetary valuation [53].

Applied to antibiotic duration, the relevant question is not whether treatment has already been beneficial, but whether further antibiotic exposure is expected to provide enough additional clinical benefit to justify its additional harms. Early in treatment, that benefit may be substantial. After effective source control, adequate antimicrobial exposure, and clinical improvement, however, the expected benefit of continuation may decline, while risks such as adverse effects, microbiome disruption, *Clostridioides difficile* infection, drug interactions, and antimicrobial-resistance selection persist. This is particularly relevant in odontogenic infections, where drainage, extraction, endodontic treatment, or debridement may substantially reduce the need for continued systemic therapy.

The concept of clinically sufficient antibiotic exposure is supported by four converging evidence streams: shorter-course trials showing non-inferiority in selected infections after adequate infection control [1,2,8,9,10,11,12,13,14]; dental evidence and guidelines emphasizing source control and reassessment [4,5,6,28,29,30,31,32,33,34,35]; PK/PD evidence showing that efficacy depends on adequate antimicrobial exposure rather than calendar duration alone [37,38,39,40]; and stewardship and ethical literature highlighting the individual and societal harms of unnecessary continuation [3,41,42,43,44,45,46,47,48,49,50,51]. In routine dental practice, antibiotic exposure is assessed clinically rather than through therapeutic drug monitoring. It is considered through antibiotic choice, dose, dosing interval, route, adherence, likely susceptibility, tissue penetration, source-control status, infection severity, host factors, and observed clinical response.

**Figure 1:**
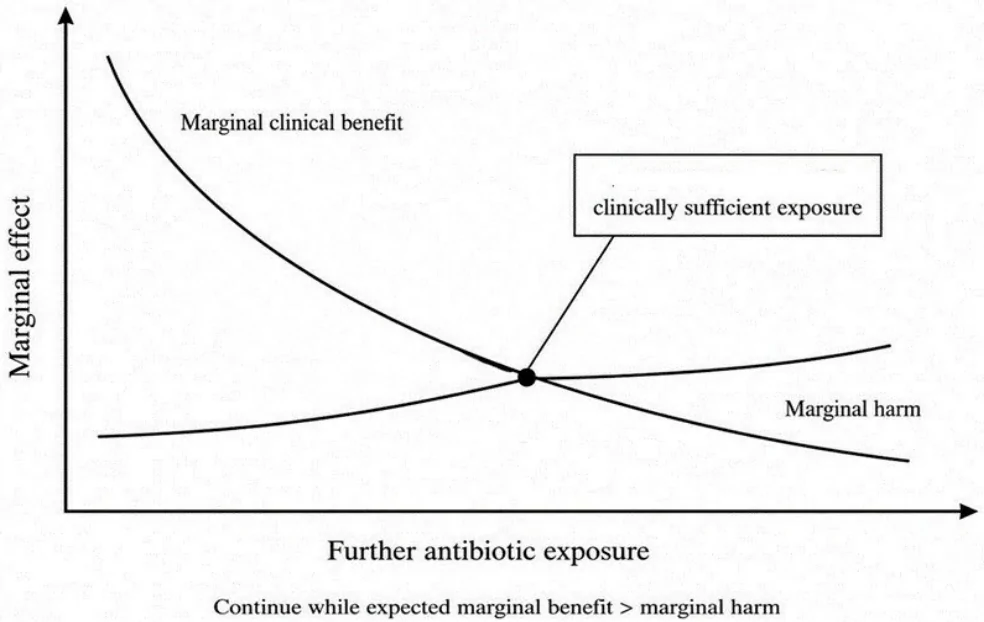
Conceptual marginal benefit–harm framework for reassessing antibiotic exposure. Early in appropriate treatment, the expected benefit of further antibiotic exposure may outweigh its harms. After effective source control and clinical improvement, that additional benefit is expected to decline. *Clinically sufficient exposure* is the patient- and infection-specific decision point at which further treatment no longer has a clearly favorable benefit–harm balance, rather than a fixed treatment duration.

Accordingly, the key question becomes: what additional clinical benefit is expected from further antibiotic exposure? Previous benefit does not, by itself, justify additional treatment. The proposed framework therefore defines clinically sufficient antibiotic exposure as the patient-specific reassessment decision point at which the expected marginal benefit of further treatment no longer clearly exceeds its expected marginal harm (Figure 1). This point is conceptual rather than numerical and depends on source control, clinical trajectory, antimicrobial adequacy, infection severity, anatomical risk, and host-related risk modifiers. The proposed checklist operationalizes this reasoning by structuring the factors that support or weaken the justification for continued exposure.

## 5. Development of the Reassessment Checklist

**Figure 2:**
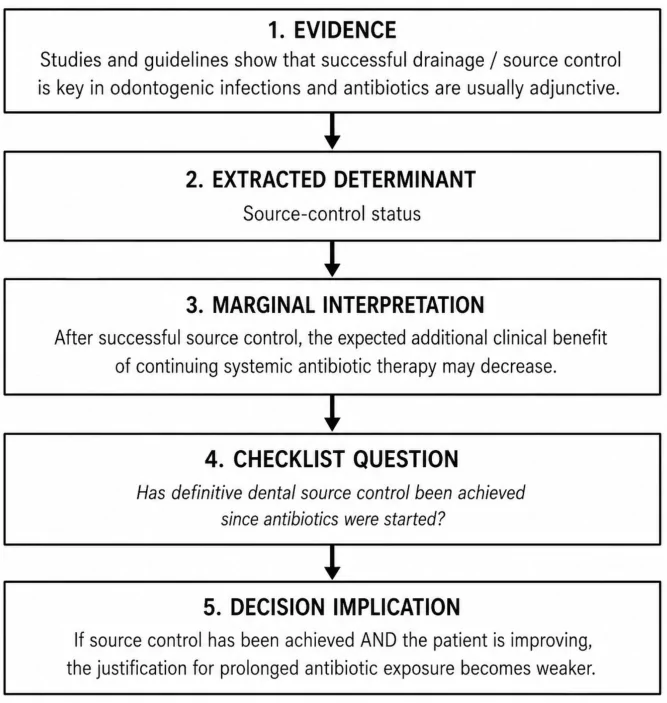
Developing the antibiotic reassessment checklist using marginal benefit–harm reasoning.

Recurring determinants relevant to antibiotic continuation or discontinuation were identified across the four predefined evidence streams and grouped into clinically relevant domains. They were then qualitatively mapped to the marginal benefit–harm framework according to the direction of effect suggested by the reviewed literature. Specifically, determinants were classified according to whether they were expected to increase or decrease the potential clinical benefit of further antibiotic exposure, or to increase its incremental harms (Figure 2). This classification was based on the authors’ conceptual synthesis of the reviewed evidence; no predefined numerical criteria, formal weighting system, or validated assignment rule was used. Persistent infection, incomplete source control, inadequate antimicrobial exposure, anatomical complexity, or host vulnerability may support continuation, whereas effective source control, clinical improvement, and adequate exposure reduce the expected benefit of further treatment. These determinants were subsequently translated into patient-level reassessment questions, so that the checklist structures but does not determine the decision whether further antibiotic treatment remains clinically and ethically justified. The checklist is therefore an evidence-informed operationalization of the framework, not a weighted score or validated decision rule. It has not yet undergone formal expert or clinical validation; future studies should assess content validity, inter-rater reliability, feasibility, and prospective clinical performance.

## 6. Checklist for Reassessing Antibiotic Duration in Dental Infection Treatment

The concept of clinically sufficient antibiotic exposure is operationalized through a structured reassessment checklist. Derived from narrative evidence mapping across antibiotic-duration studies, dental guidance, PK/PD literature, and ethical stewardship evidence, the checklist organizes the key factors that determine whether further antibiotic exposure remains justified. It does not define a numerical threshold or universal treatment duration; rather, it supports patient-specific reassessment of the expected benefit and harm of continued therapy. The checklist is intended for use at 48–72 h, at the anticipated stopping point, or when new clinical information emerges. Figure 2 summarizes the conceptual decision process, while Table 2 translates it into patient-level reassessment questions.

## 7. The Clinical and Educational Implications

Excessive antibiotic duration persists not only because evidence is unavailable or guidelines are outdated, but also because prescribing behavior is shaped by uncertainty, fear of adverse outcomes, professional norms, and reluctance to stop therapy once it has been started [36,50]. These influences can make continuation feel safer than discontinuation, even when the expected additional clinical benefit has become limited. However, direct evidence that defensive or medico-legal prescribing specifically determines antibiotic duration in dentistry remains limited, and such factors should therefore be regarded as potential contributors rather than established dental determinants [41,51]. These findings suggest that long-course prescribing functions not merely as a knowledge gap, but as a learned pattern of moral and professional self-protection: “more antibiotic” is experienced as caution, while stopping is experienced as a risk. Therefore, the educational response should not merely replace one slogan with another. Students and early-career clinicians need to learn antibiotic-duration reasoning as a core ethical and pharmacological skill: to justify duration through structured reasoning that integrates source control, clinical response, antimicrobial exposure, tissue penetration, host vulnerability, adverse effects, microbiome disruption, antimicrobial resistance, and the broader ethical consequences of unnecessary treatment [3,37,38,39,40,42,43,44,45,46,47,48]. Decision-support tools may further support this approach by helping learners practice structured prescribing decisions rather than merely retrieve guideline recommendations. Previous dental stewardship work suggests that digital educational tools can improve prescribing-related knowledge and decision-making [51]. Accordingly, such tools could potentially help learners understand that responsible prescribing is neither automatic guideline compliance nor automatic shortening, but a reasoned judgment about whether further antibiotic exposure remains clinically and ethically justified.

## 8. Limitations

This review has several limitations. First, it was designed as a narrative conceptual synthesis rather than a systematic review; therefore, the literature search was targeted rather than exhaustive, and no formal risk-of-bias or certainty-of-evidence assessment was performed. Second, the marginal benefit–harm framework and the concept of clinically sufficient antibiotic exposure are conceptual and should not be interpreted as validated clinical endpoints or numerical stopping thresholds. Since the construct has not undergone prospective clinical validation, its reliability, inter-rater consistency, predictive value, and safety as a basis for treatment discontinuation remain unknown. The proposed checklist was derived qualitatively from recurring determinants identified across the selected evidence streams and has not yet undergone formal content validation. Finally, the checklist is intended to structure patient-specific reassessment rather than replace clinical judgment or local guidelines.

## 9. Conclusions

This review proposes a conceptual reframing of antibiotic duration as a reassessment-based decision about whether further exposure remains justified, rather than as a predetermined treatment course. The marginal benefit–harm framework provides the conceptual basis for this reasoning, while the proposed checklist operationalizes it by integrating infection severity, anatomical involvement, host-related risk modifiers, and the expected incremental benefit–harm balance. In this way, the framework extends existing stewardship recommendations on short courses and reassessment by providing a structured approach to deciding how continuation or discontinuation can be justified in individual dental patients. Nevertheless, prospective evaluation is required before routine clinical implementation. 

## Figures and Tables

**Table 1:** Four evidence lines supporting the proposed framework for reassessment of antibiotic therapy duration in dentistry.

Evidence Lines	References Included	Role in the Review	Contribution to the Concept of Clinically Sufficient Antibiotic Exposure
1. Medical trials and systematic reviews comparing shorter and longer antibiotic courses	[1,2,8,9,10,11,12,13,14]	Provides the broader medical evidence that shorter antibiotic courses can be non-inferior to longer courses in selected infections when patients are clinically stable and source control is adequate.	These studies justify asking whether further antibiotic exposure is still necessary, but they do not prove that shorter therapy is always appropriate in every patient or infection type.
2. Dental reviews and guidelines on odontogenic infections, maxillofacial space infections, and antibiotic prescribing	[4,5,6,7,14,15,16,17,18,19,20,21,22,23,24,25,26,27,28,29,30,31,32,33,34,35,36]	Provides the dental-specific clinical context: source control is central in odontogenic infections. There is limited evidence base for optimal dental antibiotic duration.	High-quality dental duration trials are sparse. Short-course therapies are recommended after incision and drainage in odontogenic maxillofacial space infections.
3. Pharmacokinetic/pharmacodynamic literature explaining antimicrobial exposure	[37,38,39,40]	Explains why antibiotic efficacy depends on adequate exposure at the infection site rather than calendar duration alone.	Provides the mechanistic rationale for reframing “duration” as “exposure.” In dentistry, this is particularly important.
4. Ethics and stewardship literature on antimicrobial resistance, proportionality, and public-health responsibility	[3,18,41,42,43,44,45,46,47,48,49,50,51,52]	Provides the normative framework for antibiotic duration decisions.	Supports the ethical claim that continuation requires justification once sufficient exposure has been achieved.

**Table 2:** Proposed checklist for reassessing antibiotic duration in dental infection treatment.

Domain	Duration-Focused Reassessment Question	Possible Interpretation for Further Antibiotic Exposure	Evidence Basis
1. Initial indication revisited after 48–72 h	Was the initial indication for systemic antibiotics still clinically relevant at reassessment?	Continued exposure may be less justified if systemic signs or spreading infection have resolved and no persistent host-related factors substantially increase the risk of complications or treatment failure. Lack of improvement after 24–72 h (fever, malaise, cellulitis, trismus, dysphagia, lymphadenopathy) may support further reassessment of diagnosis, source control, adherence and antibiotic choice	Dental guidelines and stewardship recommendations [7,14,15,29,35]
2. Dental source control status	Has definitive dental source control been achieved since antibiotics were started?	If drainage, extraction, endodontic treatment, debridement, or other causal treatment has been completed and the patient is improving, prolonged therapy may offer limited additional benefit. If source control is incomplete, delayed, or uncertain, continuation may be considered only while definitive management or referral is being arranged	Dental guidance and reviews [4,5,6,7,14,15,17,18,19,20,29,31,32,35,36]
3. Clinical trajectory after 48–72 h	Is the patient clinically improving after the initial treatment period?	Reduction in swelling, pain, fever, malaise, trismus, dysphagia, lymphadenopathy, or drainage supports reassessment toward stopping at the shortest effective duration. If systemic involvement has resolved and source control is adequate, further antibiotic exposure may be difficult to justify. Current dental guidance supports discontinuing antibiotics 24 h after complete resolution of systemic signs/symptoms	Dental guidance and clinical evidence [7,14,28,29,30,31,32,33,34,35]
4. Infection severity and anatomical involvement, with host-related risk modifiers	Are there features of established infection severity, anatomical involvement, or host-related risk that may justify longer treatment?	Dose, route, interval, adherence, renal/hepatic adjustment, tissue penetration, and likely pathogen susceptibility should be reconsidered. Established clinical features such as deep fascial-space infection, airway involvement, osteomyelitis, poor drainage, or suspected spread beyond the localized odontogenic focus may directly support escalation or longer treatment. Host-related factors such as severe immunosuppression, uncontrolled diabetes, significant comorbidity, pregnancy, or frailty may modify the risk of complications or treatment failure and should be considered separately when reassessing duration	PK/PD evidence on adequate antimicrobial exposure [37,38,39,40] and dental/maxillofacial evidence on infection severity and anatomical risk [6,15,31,32]
5. Marginal benefit–harm of further antibiotic exposure	Is further antibiotic exposure expected to provide additional clinical benefit that still clearly outweighs its incremental harms?	Further exposure should be easier to justify when a specific unresolved clinical factor is present. If no clear reason for continuation can be identified, the balance may favor discontinuation, especially considering allergy, gastrointestinal adverse effects, C. difficile risk, interactions, microbiome disruption, and resistance selection	Short-course evidence [1,2,8,9,10,11,12,13,14], dental stewardship principles [7,27,35], ethical and AMR literature [3,21,22,23,24,25,26,41,42,43,44,45,46,47,48,49,50,51], and marginal-analysis reasoning [53]
6. Documentation of the duration decision	Is the reason for stopping or continuing antibiotics explicitly documented?	The reassessment note may record source-control status, clinical response, remaining risk factors, planned duration, and next review point. This supports transparent clinical reasoning rather than automatic completion of a fixed historical course	Framework-derived operational item, informed by stewardship and structured prescribing approaches [35,52]

## Data Availability

No new data were created or analyzed in this study. Data sharing is not applicable to this article.
